# Human thyroid tumours, the puzzling lessons from E7 and RET/PTC3 transgenic mice

**DOI:** 10.1038/sj.bjc.6604740

**Published:** 2008-11-04

**Authors:** L Jin, A Burniat, J-E Dumont, F Miot, B Corvilain, B Franc

**Affiliations:** 1 Institut de Recherche Interdisciplinaire (IRIBHM), Faculté de Médecine, Université Libre de Bruxelles (ULB), Campus Erasme, Route de Lennik 808, B 1070 Bruxelles, Belgique; 2 Hôpital Ambroise Paré (APHP), Université de Versailles Saint Quentin en Yvelines, Service d'Anatomie Pathologique, 9 Av., Charles de Gaulle, F-92104 Boulogne, France

**Keywords:** cribriform patterns in thyroid tumour, RET/PTC3 and E7 transgenic mice, papillary thyroid carcinoma

## Abstract

Human rearranged RET/PTC3 (papillary thyroid carcinoma) proto-oncogene and high-risk human papillomavirus (HPV) type 16 E7 oncogene induces in the mouse a neoplastic transformation of thyroid follicular cells. We present a detailed immuno-histological study (170 mouse thyroids: RET/PTC3, E7, wild type, 2- to 10-month-old) with cell cycle proliferation and signalling pathway indicators. The characteristics of both models are different. There is an ‘oncogene dependent’ cellular signature, maintained at all studied ages in the E7 model, less in the RET/PTC3 model. During tumour development a large heterogeneity occurred in the Tg-RET/PTC3 model within a same tumour or within a same thyroid lobe. The Tg-E7 model was less heterogeneous, with a dominant goitrous pattern. The solid tumour already described in the RET/PTC3 models associated with cribriform patterns, suggested ‘PTC spindle cell changes’ as in humans PTC rather than the equivalent of the solid human PTC. Proliferation and apoptosis in the two thyroid models are related to the causal oncogene rather than reflect a general tumorigenic process. The thyroids of RET/PTC3 mice appeared as a partial and transient model of human PTCs, whereas the Tg-E7 mice do not belong to the usual PTC type.

Thyroid function, growth and differentiation are mainly controlled by two pathways: the TSH-cAMP cascade and the growth factor pathways. The oncogenic stimulations of these pathways initiate different types of tumours (Dumont *et al*, 1989, 2005; Williams, 1994; Fagin, 2002; Fusco *et al*, 2002). To experimentally study these diseases, different mouse models have been created.

Rearrangements of the RET tyrosine kinase receptor gene (RET/PTCs) are found in 2.6–34% of human papillary thyroid carcinomas (PTC) and are even more prevalent in radiation-induced paediatric PTC (Baverstock *et al*, 1992; Kasakov *et al*, 1992). The rearranged RET/PTCs oncogene are activated forms of the RET proto-oncogene (Fusco *et al*, 2002; Santoro *et al*, 2002). The thyroid-targeted expression of the human RET/PTC3 oncogene under the control of the bovine thyroglobulin promoter in transgenic mice, induces metastatic solid-type papillary carcinomas considered as analogous to the human solid-type PTC (Powell *et al*, 1998).

The oncogenic properties of the high-risk human papillomavirus (HPV) type 16 E7 protein are attributed to its interaction with RB1 and related proteins (Münger *et al*, 1989; Weinberg, 1995; Coulonval *et al*, 1997). Inactivation of these proteins results in a continuous growth of the thyroid, little affecting its differentiation, function and regulation (Iuliano *et al*, 2000). There is still a debate on RB1 function in human thyroid tumorigenesis with contradictory results on its loss or overexpression in papillary and follicular carcinomas *vs* adenomas (Anwar *et al*, 2000).

The mice expressing the human original HPV type 16 E7 protein under the control of the thyroglobulin promoter develop differentiated and functionally regulated thyroid goitres, before the occurrence of tumours mimicking human-differentiated follicular and papillary thyroid carcinomas. In these transgenic mice, by 1 month, follicular cells are modified, small, and numerous with an increased nuclear/cytoplasmic ratio (Ledent *et al*, 1995; Coppée *et al*, 1996). Tumours appearing, over 1 year, have been compared with human thyroid insular carcinomas (Franc *et al*, 1998).

The coexpression of RET/PTC 3 and E7 in transgenic mouse models lacks any cooperative effect in the neoplastic transformation of thyroid cells and the E7-induced thyroid phenotype is dominant with respect to the RET/PTC3 one. These results are intriguing and were poorly explained (Portella *et al*, 2000).

Homologies between the E7 model and several features encountered in human benign and malignant thyroid disease have been little explored. We know much about the pathology of RET/PTC human tumours but much less about their biological evolution. The meaning of solid patterns in human thyroid tumours are a matter of debate.

We have therefore conducted a parallel study of the evolution of gene expression and pathology in E7 and RET PTC3 mice. The transcriptome study (Burniat *et al*, 2008) revealed differences of gene expression between E7 and RETPTC3 models and between different phases in the evolution of the tumour. The pathological and immunohistological study presented here, in which thyroid function, proliferation, and the two involved signalling pathways were completely tested and they explain the gene expression results. Our results permit to further explore some of the diagnostic enigma in human thyroid pathology.

## Materials and methods

RET/PTC3, E7 transgenic and C57Bl/6 wild-type mice were fed with a normal iodine diet and maintained in the animal facility under 12 h light/12 h darkness cycle. Animal studies were approved by the Animal Care Use and Review Committee (Commission d'éthique du Bien–Etre Animal) of the Faculty of Medicine, Université Libre de Bruxelles (project 160N, 2007–2010) and in compliance with the UKCCCR guidelines on animal use.

Experimental groups consist of E7 mice (*n*=87), RET/PTC3 mice (*n*=53) and the C57Bl/6 mice (*n*=30) served as controls. RET/PTC3 and E7 transgenic mice provided by Dr M Santoro (Napoli) and Dr C Ledent (our Institute) respectively were crossed with wild type C57Bl/6. Both strain progenies were genotyped by PCR (Burniat *et al*, 2008).

Genomic DNA was prepared from a piece (0.5 cm) of tail by a lysis treatment at 55°C in TNES (100 mM Tris-HCl pH 8/200 mM NaCl/5 mM EDTA/0.2% SDS/40 *μ*g proteinase K). Selected regions of the human RET and E7 genes were amplified by the PCR method. The primers used for the amplification were: RET forward, 5′-GGCCAGAGCCCTAAGGAGGGC-3′; RET reverse, 5′-AAGGGATTCAATTGCCATCCA-3′. E7 forward, 5′-CATGCATGGAGATACACCT-3′; E7 reverse, 5′-GATTATGGTTTCTGAGAACA-3′. Amplified products were visualized on agarose gel as 1500 and 300 bp bands respectively.

The antibodies used to test thyroid function were: anti-Tg polyclonal antibody (Dako, Glostrup, Denmark), anti-Tg-I monoclonal antibody B1 (gift from Dr C Ris-Stalpers), anti-NIS rabbit polyclonal antibody (gift from Dr N Carrasco), cell proliferation and signalling pathways: anti-BrdU monoclonal mouse antibody (Becton Dickinson, Rutherford, NJ, USA), anti-Ki-67 monoclonal rat antibody (clone TEC-3, Dako), anti-Cyclin D1 monoclonal rabbit antibody (SP4, NeoMarkers, Fremont, CA, USA); anti-Phospho-p44/42 MAPK Thr202/Tyr204 monoclonal rabbit antibody (20G11, Cell Signalling, Danvers, MA, USA), anti-Phospho-Akt Ser473 monoclonal rabbit monoclonal antibody (736E11, Cell Signaling). Primary antibodies, dilution and incubation time are listed in Table 1.

### Immunohistochemistry, radioiodide uptake, and proliferation index determination

The transgenic and control mice were killed at 2, 6 and 10 months of age and thyroids were removed for pathological examination. One or both lobes were fixed in 4% paraformaldehyde and embedded in paraffin by standard procedures.

For determination of radioiodide uptake, mice were injected intraperitoneally (i.p.) with 0.5 mCi kg^−1^ body weight Na^125^I (IMS 30; PerkinElmer Life and Analytical Sciences, Boston, MA, USA) in physiological serum. Twenty-four hours after ^125^I injection, they were perfused with 0.9% NaCl during 15 min before killing. After deparaffinisation and rehydratation, the slides were dipped two times in 42°C NTB3 Emulsion (Kodak), stored in light tight box at 4°C for 8 days, developed with Kodak D-19 Developer and stained with haematoxylin–eosin (H&E).

For determination of the proliferation index, 5-Bromo-2′-deoxyuridine (BrdU, Sigma, Saint Louis, MO, USA) 0.5 mg g^−1^ body weight was injected i.p. 24 h before killing. The thyroids were fixed in Clarke's fixative (75% V/V ethanol: 25% V/V glacial acetic acid) 18 h after perfusion of the mice, embedded in paraffin. BrdU was detected by immunohistochemistry. DNA in the samples was denatured in two N HCl, followed by neutralization in 0.1 M borate buffer, pH 8.5.

For immunohistochemical analysis of protein expression, the endogenous peroxidase activity was quenched by a treatment with 6% H_2_O_2_ in absolute methanol; non-specific-binding sites were blocked with 10% normal goat serum for Tg and Tg-I, normal horse serum for others. After incubation with primary antibodies, tissue sections were sequentially incubated with second antibodies conjugated to peroxidase-labelled polymer (EnVision detection, Dako) for Tg and Tg-I or goat biotinylated secondary antibodies (Jackson ImmunoResearch, West Grove, PA, USA) (Table 1) and Avidin-conjugated horseradish peroxidase ABC system (Dako) for others. Staining was developed with aminoethylcarbazole-AEC (Dako) and diaminobenzidine-DAB (Dako) substrates (Table 1) and sections were slightly counterstained with Mayer's haematoxylin. Negative controls were performed using a replacement of the first antibodies with a blocking solution.

The BrdU, Ki-67, and Cyclin D1 labelling index values were evaluated by counting the number of immunopositive cells out of a total of thousand cells (Mean±s.e.m.). At least two different slides from the same mouse thyroid were counted.

## Results

The thyroids of all the mice used presented a similar level of E7 or RET/PTC mRNA transgene expression by RT–PCR (not shown). When compared with the wild type (WT) taken as control, RET/PTC3 and Tg-E7 transgenic mice thyroid harboured two different phenotypes. According to age, the Tg-E7 model evolved on a predominant goitrous mode with no tumour formation, whereas tumour formation occurred in 28% of the RET/PTC3 mice at 6 and 10 months. A hyperplastic background occurred in both models with peculiar characteristics in the RET/PTC3 model.

The dominant feature at the cellular level was the occurrence of ‘oncogene-dependent’ follicular cell changes, leading to different cellular phenotypes, small cells in the E7 model and large cells in the RET/PTC3 model, which evolved differently with time.

In the E7 model these cellular changes were not reminiscent of the human PTC cells, which are also different from the RET/PTC3 model.

Those histological findings were accompanied by changes in the expression of proteins related to thyroid function, proliferation indices, PKB and MAPkinase pathways.

Our results represent a precise histological description of the follicular cell changes, hyperplastic and tumour features, followed by corresponding data concerning thyroid function, cell proliferation, PKB and MAPkinase pathway indicators. The several encountered modifications occurring in the transgenic models in comparison with controls are described according to age.

### Follicular cell changes

**Figure 1:**
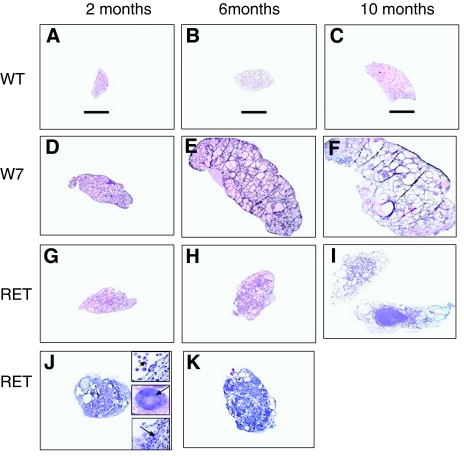
Paraformaldehyde-fixed, paraffin-embedded and haematoxylin–eosin stained thyroid tissues from C57Bl/6, E7 and RET/PTC3. (**A**–**C**) 2, 6, and 10-month C57Bl/6 thyroids respectively; (**D**–**F**) 2-, 6-, 10-month E7 thyroids respectively; (**G**–**I**) 2-, 6-, 10-month RET/PTC3 respectively, (**I**) two lobes from the same mouse; (**J** and **K**) 2-, and 6-month RET/PTC3 with remodeling and cyst (diffuse sclerosing); upper right insert of **J**: macrophages infiltration, middle right insert of **J**: nuclear inclusion; low right insert of **J**: thick spindle-like cells in between follicles. Original magnification 2.5 × . Scale bar: 1 mm.

**Figure 2:**
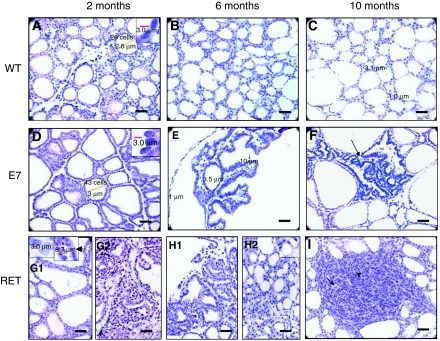
Haematoxylin–eosin stained C57Bl/6, E7 and RET/PTC3 thyroid glands. (**A**–**C**) wild type C57Bl/6 thyroids; (**D**–**F**) E7 thyroids; (**G**–**I**) RET/PTC3 thyroids. Upper right inserts in **A**: WT nuclei, in **B**: E7 nuclei and upper left insert in **G1**: pale, pleomorphic with dispersed chromatin nuclei and nuclear groove, red scale bar: 3 *μ*m (100 × ), upper right insert in **G1**: large thyrocytes with a decreased ratio of nuclear/cytoplasm (0.27–0.55) compared with WT (1.20) and E7 (3.97–22.67) thyrocytes, red bar equal to 8.7 *μ*m (40 × ); (**G2**) 2-month RET/PTC3 with remodeling and cyst (diffuse sclerosing); (**H1**) disorganised papillary cyst zone in 6-month RET/PTC3 thyroid. (**H2**) cribriform structures in RET/PTC3; upper insert: two small follicles share one part of the follicular ‘wall’; (**I**) solid tumour structure in RET/PTC3, long arrow: cells harboured a large cytoplasm and oval nucleus, short arrow: spindle-like with less cytoplasm. Original magnification × 40 for **A**–**I**; scale bar: 30 *μ*m.

At 2 months, WT mouse thyroids harboured cuboidal follicular cells lining small follicles (Figures 1A–C and 2A). The size of the follicles became heterogeneous at 6 months, with dominant large follicles lined by flat cells at 10 months (Figure 2B and C).

**Figure 3:**
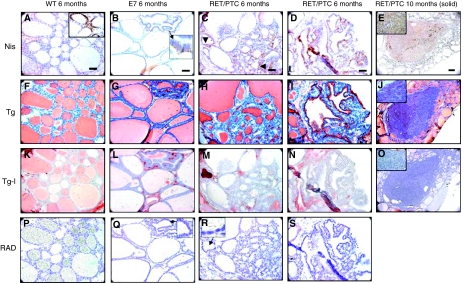
Immunohistochemistry for Sodium Iodide Symporter (NIS), Thyroglobulin (Tg), Iodinated-thyroglobulin (Tg-I) and incorporation of iodide visualized by autoradiography (ARG). (**A**, **F**, **K** and **P**) 6-month C57Bl/6, NIS, Tg, Tg-I immunostaining and autoradiography, respectively; (**B**, **G**, **L** and **Q**) 6-month E7, NIS, Tg, Tg-I immunostaining and autoradiography, respectively; (**C**, **H**, **M** and **R**) 6-month RET/PTC3, NIS, Tg, Tg-I immunostaining and autoradiography, respectively; (**D**, **L**, **N** and **S**) 6-month RET/PTC3, disorganized papillary cyst region NIS, Tg, Tg-I immunostaining and autoradiography respectively; (**E**, **J** and **O**) 10-month RET/PTC3, solid mass region NIS, Tg, Tg-I immunostaining, respectively. Original magnification for (**E**, **J** and **O**): × 10, scale bar: 100 *μ*m. Original magnification for other images: × 40; scale bar:30 *μ*m. Inserts magnification: × 40.

E7 thyroids were much larger than WT thyroids at all ages (Figure 1D–F). At 2, 6, and 10 months, the follicular cells, with a not well marked cellular border, were crowded and overlapping all around the follicles, with a 1.9-fold cell number increase at 2 months in comparison with the WT (Figure 2A and D). The nuclei were hyperchromatic with an increase in the nuclear/cytoplasmic ratio (Figure 2D), The follicular border appeared ‘palissade like’ (Figure 2D–F). At 6 and 10 months huge follicles lined with flat follicular cells (Figures 2E, F, 3B and Q) coexisted with small round and irregularly shaped follicles with ‘palissade like’ borders (Figures 2E, F, 3B and Q).

RET/PTC3 thyroids were larger than control thyroids at all ages but smaller than those of the E7 model (Figure 1). The modifications were different from one mouse to another.

At 2 months, part of the cells were modified; they were larger (more than 8 *μ*m instead of 3 *μ*m), lining all or part of the thyroid follicles, stratified in some areas (Figure 2G). The cytoplasm was large with a convex apical border and a decreased nuclear/cytoplasmic ratio, a pale nucleus, sometimes pleomorphic with dispersed chromatin (Figure 2G). The nucleus location and polarity were disturbed (Figure 2G). At 6 and 10 months, many follicular cells were still large but some were not (Figure 2H and I).

### Hyperplastic features

Absent in the WT, hyperplastic areas were present in both E7 and RET/PTC 3 as micro or larger papillary infoldings with or without secondary follicles. Present already in the 2-month models, these papillae became more numerous with age, especially in the E7 model with a branching distribution, often situated in the centre of the lobe inside dilated follicles (Figure 2D–F). In the RET/PTC3 model, the thyroid organisation was progressively replaced at 6 and 10 months, in 20% of the examined thyroids, by a cribriform pattern difficult to distinguish in some cases from a true diffuse tumour process (Figure 2H2). The cell changes already described persisted in the two models at 6 and 10 months.

In 28% of the examined RET/PTC3 thyroids a peculiar pattern emerged at 2 months. Thyroid lobes were totally replaced by a series of large cysts filled with papillary structures more or less coalescent, bordered with large cells, with rare nuclear cytoplasmic pseudo inclusion (Figure 1J middle insert) and pleomorphism. Peripheral cysts were filled with macrophages (Figure 1J). A mixture of true mesenchymatous tissue and sheets of spindle or more round cells, in close vicinity with the wall of the cyst or their papillary content, existed between cysts (Figures 1J and 2G2). In the absence of a true definition of this pattern in the literature, we identify them as ‘proliferative papillary cystic changes with spindle cells and remodeling’. They were rare at 6 months and absent at 10 months.

Peripheral large cysts with a discrete micropapillary border in an otherwise ‘normal’ or moderately hyperplastic thyroid structures were encountered in 45 and 58% of the 6 and 10 months thyroid RET/PTC 3 models respectively (Figures 2H1, 3D, I, N and S).

### Distinct tumours

They were absent in the WT and we did not find any in the E7 model. In the RET/PTC3 mice, true tumours were already visible in 16% of the cases, either at 6 months and most often at 10 months (Figure 1I). These tumours (small foci around 1 mm or macroscopically visible 5 mm diameter tumour) were unique or multiple. They were not or only partially circumscribed. The dominant histological pattern was solid without an obvious follicular or papillary structure. Cells harboured either a large cytoplasm (Figure 2I) and oval nucleus or were spindle-like with less cytoplasm (Figure 2I). Some mitoses were observed. The two thyroid lobes of the same mouse could show different lesions, mostly associated with the ‘cribriform pattern’ (Figures 1I and 2H2).

### Thyroid function

#### WT mice

At all ages Tg staining was observed in the follicular lumen (Figure 3F). Radioiodine incorporation (Figure 3P) paralleled the immunostaining of Tg-I (Figure 3K). At 2 months the follicles presented a high capability to bind radioiodide, which decreased and disappeared in the follicles lined with flat cells at 6 and 10 months (Figure 3K and P). NIS was detected in a baso-lateral position of the follicular cells at 2 months (not shown). This uniformity decreased with age and NIS was absent in the flat cells (Figure 3A).

#### E7 mice

At all ages Tg staining was observed in the follicles (Figure 3G). Half of the follicles were labelled at 2 months for Tg-I but the corresponding staining decreased at 6 and 10 months (Figure 3L and Q). Tg-I persisted in the small round and irregular shaped follicles and in the follicles close to the papillary infoldings (Figure 3L). Autoradiography confirmed the limited localisation of Tg-I (Figure 3Q). NIS was detected in the majority of thyroid epithelial cells at 2 months and in the Tg-I-positive areas at 6 and 10 months. It disappeared in the flat cells (Figure 3B).

#### RET/PTC3 mice

At 2 months, NIS was expressed only in the large cells. The number of follicles positively labelled for Tg-I and displaying radioiodine was systematically lower than in E7 and WT mice. Tg was observed in all follicles. By 6 months and later, low cuboidal-flat follicular cells displayed a rare expression of NIS, no radioactive iodide incorporation (Figure 3C and R) and no Tg-I immunostaining (Figure 3M) whereas Tg was still present (Figure 3H). In the disorganised papillary regions at the periphery of the gland, the modified follicular cells expressed NIS in a baso-lateral position (Figure 3D). The lumen was devoid of iodide uptake and Tg-I labelling. Tg only persisted as a lining at the apical border of the follicular cells (Figure 3I, N and S).

In the solid tumour, NIS and Tg were situated in the cytoplasm of the cells. The intensity of the staining varied from one region to the other (Figure 3E and J). Tg-I was negative (Figure 3O).

### Thyroid tumour growth

**Figure 4:**
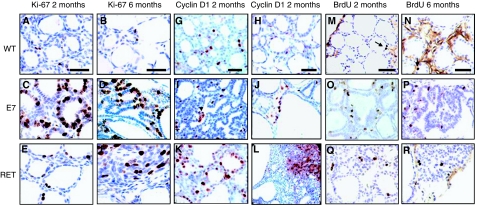
Immunohistochemistry for Ki-67 (**A**–**F**), Cyclin D1 (**G**–**L**) and BrdU stainings (**M**–**R**) in thyroid tissues from C57Bl/6, E7 and RET/PTC3. (**A**, **G** and **M**) 2-month C57Bl/6; (**B**, **H** and **N**) 6-month C57Bl/6; (**C**, **I** and **O**) 2 month E7; (**D**, **J** and **P**) 6-month E7; (**E**, **K** and **Q**) 2-month RET/PTC3; (**F**, **L** and **R**) 6-month RET/PTC3. Upper left part of (**F**) and upper right corner of (**L**): solid mass regions. (**R**): disorganized papillary tumour region. Original magnification: × 40; scale bar: 30 *μ*m.

Measurements of Ki-67 and BrdU-labelling indexes showed the same features. In WT, RET/PTC3, and E7 thyroids the overall proliferation index was the highest at 2 months and decreased with age. Compared with WT, the frequency of positive proliferation indexes was systematically higher in both thyroid models and higher in E7 than in RET/PTC3 at all ages (Table 2). In 2-month-old E7 thyroids, the number of proliferating cells was increased compared with WT (Table 3, Figure 4C and O) and homogenously distributed. At 6 and 10 months, proliferating foci were mainly located in small irregularly shaped follicles and papillae. Proliferation was very rare in flat cells (Table 3, Figure 4D and P).

In RET/PTC3 thyroids proliferating foci were often located in the papillary infoldings and in the solid tumour (Table 3, Figure 4F). Cyclin D1 immunolabelling paralleled the results obtained with BrdU and Ki-67 (Table 3, Figure 4K and L).

**Figure 5:**
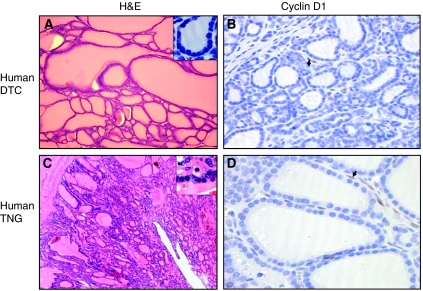
Haematoxylin–eosin and Cyclin D1 immunohistochemistry stainings of **A** and **B**: human differentiated thyroid carcinoma; (**C** and **D**) Human thyroid nodular goitre. Upper right inserts in **A** and **C**: small thyrocytes with high nuclear/cytoplasm ratio. Arrows in **B** and **D**: positive stromal cell nuclei for Cyclin D1. Original magnification: × 20 for **A**; × 60 for **B**; × 4 for **C** and × 40 for **D**.

In the E7 model, Cyclin D1 immunolabelling was very low in follicular cells compared with WT, but was positive in the nucleus of a great number of stromal cells. It was rarely positive in control stroma (Table 3, Figure 4I and J). Some human thyroid nodular goitre and differentiated thyroid carcinoma presenting morphology and nuclear features (Figure 5A and C) similar to E7 mouse model also showed stromal cell-positive staining for Cyclin D1 (Figure 5B and D). In mouse E7 model, no apoptosis, as measured by Tunel assay and by immunohistochemical detection of activated caspase 3, could be observed in thyrocytes of both transgenic models and wild-type mice (not shown).

### The PKB and MAP kinase pathways

**Figure 6:**
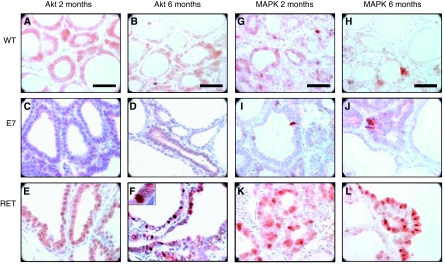
Immunohistochemistry for p-PKB/Akt (**A**–**F**) and p-MAPK (**G**–**L**) in thyroid tissues from C57Bl/6, E7 and RET/PTC3. (**A** and **G**) 2-month C57Bl/6; (**B** and **H**) 6-month C57Bl/6; (**C** and **I**) 2-month E7; (**D** and **J**) 6-months E7; (**E** and **K**) 2-month RET/PTC3; (**F** and **L**) 6-month RET/PTC3. Original magnification: × 100; scale bar: 30 *μ*m.

At 2 months, phosphorylated PKB/Akt expression was cytoplasmic, slightly weaker in E7 (Figure 6C and D) than in RET/PTC3 (Figure 6E and F) and WT mice (Figure 56A and B). Staining decreased at 6 months in WT thyroid, but was unchanged in RET/PTC3 follicular cells (visible in some nuclei) (Figure 6B and F).

In WT thyroids phosphorylated MAPK showed a nucleo-cytoplasmic staining that almost disappeared at 6 months (Figure 6G and H). In RET/PTC3 MAPK staining was stronger and predominantly located in the nucleus. It was maintained at 6 months (Figure 6K and L). On the contrary, only a weak labelling was found in the cytoplasm of E7 epithelial cells with the ‘palissade like’ appearance and sometimes in their nucleus (Figure 6I and J).

## Discussion

As in previous studies on transgenic E7, and RET/PTC3 mouse models (Ledent *et al*, 1995; Coppée *et al*, 1996; Powell *et al*, 1998; Portella *et al*, 2000) the generally encountered features such as hyperplasia, and solid tumours in the RET/PTC3 model are present. The cellular modification and the goitre present in the E7 model were as already described. However, we do not find tumours in the E7 model, but our study ended earlier (10 months) than the other studies. We do not find in the literature any precise description of the cellular modification encountered in the RET/PTC3 model, nor fully documented data on the several tested parameters we presented (thyroid function, proliferative indices, pathway indicators). Some studies tested thyroid function, as serum thyroid hormone level, or tissue thyroglobulin expression (Ledent *et al*, 1995). Few of the tested cell cycle indicators presented in our study have been tested. Moreover our findings on cyclin D1 expression in an E7 model have never been previously mentioned. In both models the PKB and MAPkinase pathways were not analysed. However, the low rate of tumour formation encountered in our study of the E7 or RET/PTC3 models differed from previous studies. In our E7 model, at 10 months, we do not find tumours, whereas a 15% occurrence of carcinomas at 12 months was observed in the E7 model (Portella *et al*, 2000). In the RET/PTC3 model, Portella *et al* (2000) described 42% of tumours at 12 months, Powell *et al* (1998), 55% at 6 months.

To explain such discrepancies, we can exclude a loss of expression of the transgene. Its expression was constant from one animal to the other and did not vary with age. Nevertheless, within one thyroid, one could not assert a uniformity of expression of the transgene all over the thyroid. The homology of the studied strains in the different literature series must be discussed. A long time duration exists between the generation of the two transgenic mice models and our study. Moreover the initial hybrid background present in both models, had declined with time due to a backcross in our study with a C57Bl/6 wild type. This can probably explain part of the differences with older series. This backcross should represent a good opportunity to identify modifier genes in the C57B1/6 genetic background responsible for the phenotype changes. In addition, we cannot exclude terminological differences as part of our discrepancies.

Our extensive study raises several points:

- the association in both models of peculiar cellular dysplastic changes and hyperplasia which could be called ‘dysplastic hyperplasia’.
- the heterogeneity we describe in our RET/PTC3 model with the cribriform patterns and the ‘proliferative papillary cystic changes with spindle cells and remodeling’ is not mentioned by others. However, the picture obtained in our study corresponds to some of the descriptions by Powell *et al* (1998); Powell *et al* (1998) define PTC ‘as cellular nodules adjacent to, or extending into colloid filled follicles’; Russell *et al* (2000) define carcinoma as: ‘more than 50 thyroid follicular epithelial cells clustered together with a homogeneous appearance. Epithelial cell clusters may form a continuous sheet or papillae that surround a fibro-vascular stalk. The nucleus may be either homogeneous or pleiomorphic’. No precisions are added considering whether or not those cell proliferations are inside a follicle. In human pathology, a diagnosis of thyroid carcinoma would not be accepted on such criteria, for example when the proliferation is included in a follicle, even if distended.

A similar confusion arises when the solid-type papillary carcinoma occurring in RET/PTC 3 transgenic mice is considered as an equivalent of the solid PTC variant found in radiation-induced thyroid cancer of children harbouring an RET/PTC3 rearrangement (Powell *et al*, 1998; Thomas *et al*, 1999). The encountered mouse features are very far from the solid human variant in which the tumour cells with PTC nuclear features are still round, organised in sheets or trabeculae of variable size, with a tendency to keep follicular patterns, even rudimentary.

The solid pattern described in transgenic mice is not specifically induced by RET/PTC3 oncogene. Several transgenic mouse models, either RET/PTC1 (Jhiang *et al*, 1996), RET/PTC3 (Powell *et al*, 1998; Portella *et al*, 2000), TRK-T1 (less often) (Russell *et al*, 2000), A2aR/E7 (Coppée *et al*, 1996) and so on, develop solid tumours. Certain conditions favoured the occurrence of a solid phenotype: TSH stimulation of RET/PTC1 model (Sagartz *et al*, 1997), double transgenic mice RET/PTC3 P53−/53− (La Perle *et al*, 2000; Powell *et al*, 2001). Other conditions prevent it such as the double transgenic model RET/PTC3-E7 (Portella *et al*, 2000).

The RET rearrangement, either RET/PTC1 or RET/PTC3, looks as a facilitator in the development of spindle cells in transgenic mice at early stage (Cho *et al*, 1999), or later on (Williams, 1994; Sagartz *et al*, 1997). The association with a cribriform pattern is also mentioned in the RET/PTC1 mouse model (Jhiang *et al*, 1996). Pictures from RET/PTC3 and RET/PTC1 solid variants show some differences; large proliferative spindle cell areas with less visible cytoplasm are more developed in the RET/PTC3 model (Sagartz *et al*, 1997; Powell *et al*, 1998).

In the human series of Nikiforov *et al* (1997), RET/PTC3 tumours are solid, PTC classical, follicular, or a diffuse sclerosing variant. In the adult series of Basolo *et al* (2002), one-third is tall cell PTC, as in part of the Nakazawa *et al* (2005) PTC series. We cannot precisely determine whether or not our solid mouse tumour corresponds to a PTC with a spindle-cell metaplasia as described in human PTC (Vergilio *et al*, 2002) or to a progressive transformation in a less differentiated phenotype (Bronner and LiVolsi, 1991; Brandwein-Gensler *et al*, 2004). Favouring this last hypothesis, the morphology of these solid tumours is heterogeneous, unlike the homogeneous description mentioned in early literature (Powell *et al*, 1998). Indeed in these areas one observes a modified NIS, Tg, and TTF-1 immunolabelling and high proliferation indices, as in human aggressive tumours.

The association of cribriform tumour patterns and spindle cells is described in the columnar human PTC (Wenig *et al*, 1998) without any knowledge of the RET/PTC3 status, and in the morular/cribriform PTC (Harach *et al*, 1994; Cetta *et al*, 1999). This last entity, sporadic or associated with the Familial Adenomatous Polyposis syndrome, has been studied at the molecular level, which revealed an APC germline mutation associated with somatic RET/PTC3 oncogene rearrangement (Soravia *et al*, 1999).

‘The proliferative papillary cystic changes with spindle cells and remodeling pattern’, which emerged at a quite early age of 2 months in our RET/PTC3 model is puzzling. The number of such phenotypes decreased at 6 months and totally disappeared at 10 months. We wonder whether it may correspond to the rare human young age ‘diffused sclerosing’ variant (Chow *et al*, 2003), with its mesenchymal remodeling and inflammatory background, or as found by Russell *et al* (2004) in the RET/PTC3 mouse in the strain background C57 BL/6.

In both RET/PTC3 and E7 transgenic mouse thyroids, the ability to concentrate radioiodide is generally reduced as in human thyroid carcinomas (Gérard *et al*, 2003).

In the disorganised papillary cystic regions of the RET/PTC3 model, NIS is overexpressed, but not Tg-I, and there is no iodide uptake. This can be correlated with data in the human PTC. It could be due to an increased intracellular NIS misexpression (Saito *et al*, 1998; Wapnir *et al*, 2003; Riesco-Eizaguirre and Santisteban, 2006), to conformational changes (Gérard *et al*, 2003) or to the non maturation of hNIS (Trouttet-Masson *et al*, 2004).

RET stimulates MAPK and Akt/PKB-signalling pathways, through Ras-Raf-Merk-Erk, Ras-PI3-K/Akt/PKB *in vivo* and vitro (Melillo *et al*, 2005; Mitsutake *et al*, 2006; Santoro *et al*, 2006). Activated Akt/PKB prevents apoptotic death in many cell types (Brunet *et al*, 1999). This probably explains why almost no apoptotic cell was detected in our RET/PTC mice.

In RET/PTC3 mouse thyroids, a strong labelling of phosphorylated MAPK in nuclei of the large cells and the cells in solid mass suggests a marked stimulation of the MAPK pathway. It parallels the same findings in the rat RET/PTC3-transfected thyroid cell line (Basolo *et al*, 2002).

Apart from these pathological features the transgenic models raise several interesting functional points. Double transgenic mice of RET/PTC3 and E7, and A2R and E7 showed that E7-mediated thyroid phenotype is dominant (Coppée *et al*, 1998; Portella *et al*, 2000). E7 oncogene acts downstream of RET/PTC3, and directly binds and inhibits RB1 protein and/or related proteins, therefore relieving their inhibition of transcription factors E2Fs (Ledent, 1996; Dyson, 1998; Helin, 1998). This could explain the scarcity of thyrocytes nuclei positive for Cyclin D1. It suggests that Cyclin D1 expression in thyroid epithelium of the E7 cells is inhibited by a negative feedback of the Cyclin D1-RB1-E2F1 axis. The cyclin D1 staining of stromal cells (not seen in WT and RET/PTC3 thyroids) is puzzling and not reported in human thyroid carcinomas (Wang *et al*, 2000). However, we have identified such features in human thyroid goitres, and in malignant tumours with ‘E7 like’ cellular features. It suggests that stromal cells get direct or indirect autocrine or paracrine signals from E7-expressing thyroid epithelium.

The activation level of the MAP kinase and Akt/PKB was different between E7 and RET/PTC3 trangenic models. E7 acts downstream of these pathways and therefore does not activate them. E7 relieves the cell of any necessity of RB phosphorylation by cyclin-dependent kinases CDK. PKB/Akt cytoplasm staining evokes the maintenance by an unknown mechanism of a weak phosphorylation, which might prevent apoptosis.

Differences between thyroid cancer prone mouse strains have been demonstrated by others (Knostman *et al*, 2007). In our thyroid transgenics this phenomenon might explain strain-to-strain alterations (Russell *et al*, 2004; Knostman *et al*, 2007) as well as a detectable background of thyroid neoplasia.

Nevertheless the models allow a kinetic *in vivo* model of the process of tumorigenesis by the oncogenes. Gene expression study by microarray technology has shown that important biological processes are differently regulated in the two tumour models: in E7 thyroid cells overexpression of cell cycle genes constitutes the most upregulated process whereas RET PTC3 thyroid cells present an upregulation of human PTC markers, of EGF-like growth factors, and extracellular remodeling genes (Burniat *et al*, 2008). Similarities with human PTC are observed at a young age but are incomplete. Therefore RET/PTC3 tumours are partial and transient models of human PTC. These results are now confirmed at a histological level with two different morphofunctional phenotypes: one with small cell looking like both primordial and differentiated, the other with large cells. A later study of gene expression in laser-microdissected areas will allow to characterize the molecular phenotype of these variants.

## Figures and Tables

**Table 1:** Detection of thyroid-specific, cell cycle and cell pathway proteins

**Antibodies**	**Incubation time**	**Dilution**	**Second antibody**	**Revelation substrate**
Nis	Overnight	1/40 000	Polyclonal Ab anti-Ig-HRP	DAB
Tg	3 h	1/3000	EnVision rabbit	AEC
Tg-I	Overnight	1/2000	EnVision mouse	AEC
Ki-67	Overnight	1/150	Polyclonal Ab anti-Ig-HRP	AEC
BrdU	Overnight	1/50	Polyclonal Ab anti-Ig-HRP	DAB
CyclinD1	Overnight	1/150	Polyclonal Ab anti-Ig-HRP	AEC
MAPK	Overnight	1/125	Polyclonal Ab anti-Ig-HRP	DAB
Akt	Overnight	1/80	Polyclonal Ab anti-Ig-HRP	AEC

AEC=3-amino-9-ethylcarbazole; DAB=3,3′-Diaminobenzidine tetrahydrochloride.

**Table 2:** General table of dominant patterns in RET/PTC3 thyroids

**RET/PTC3**	***n***	**Mild change (MD)/hyperplasia**	**Papillary cystic proliferative changes with spindle cells and remodelling hyperplasia**	**Hyperplasia and solid tumour**	**Cribriform patterns**	**Cyst with micropapillae**
2 months	21	8 (MD)(38%)/7 (33%)	6 (28%)	0	0	0
6 months	20	8 (40%)	1 (5%)	2 (10%)	2 (10%)	9 (45%)
10 months	12	1 (8%)	0	3 (25%)	2 (16%)	7 (58%)

**Table 3:** Proliferation index in E7 and RET/PTC3 thyroid cell

**E7 (*n*=83)**						
**Age of mice**	**2 months (*n*=26)**	**6/10 months (*n*=57)**				
**Pathological changes**	**Hyperplasia**	**Papillae and small irregular follicles “palisade like” border**	**Follicles cuboidal cell**	**Large follicles: flat cell**		
**Height of follicular cells (*μ*m)**	**3.0 *μ*m**	**10.0 *μ*m**	**3.0 *μ*m**	**1.0 *μ*m**		
*Cell cycle markers*						
Ki-67	368.19‰ (WT 17.27‰)	317.70‰ (WT 8.26‰)	67.20‰ (WT 8.26‰)	8.20‰ (WT 8.26‰)		
Cyclin D1						
Thyrocytes	0.143‰(WT 11.1‰)	0.275‰(WT 2.25‰)				
Stromal cells	21.81‰	33.79‰	10.55‰	3.87‰		
BrdU	44‰ (WT 3.7‰)	14.23‰ (WT 0.4‰)	5.29‰ (WT 0.4‰)	0.43‰ (WT 0.4‰)		
						
						
**RET/PTC3 (*n*=53)**						
**Age of mice**	**2 months**		**6/10 months**			
**Pathological changes**	**Mild change/hyperplasia**	**Papillary cystic with spindle cells and remodeling**	**Mild changes**	**Hyperplasia, cribriform patterns**	**Cysts with micropapillae**	**Solid mass region**
**Height of follicular cells (*μ*m)**	**1.6-8.9 *μ*m**	**1.6-8.9 *μ*m**	**1.6-3.1 *μ*m**	**3.1-8.9 *μ*m**	**3.1-8.9 *μ*m**	NE*
*Cell cycle markers*						
Ki-67	29.96‰	86.01‰	3.98‰	30.28‰	33.08‰	62.44‰
Cyclin D1	95‰	220‰	1.6‰	20‰	201‰	203‰
BrdU	7.8‰	34‰	0.6‰	7.0‰	13‰	NE

NE=not examined. NE^*^ the heterogeneity of the cellular component (size and shapes) does not permit any measurement.

The proportion of epithelial cells labeled was estimated as per thousand (‰) of the total number of thyrocytes, and presented as mean values±s.e.m. For scoring, at least a thousand thyrocytes on two different slides were counted in one mouse thyroid.
