# Human thyroid tumours, the puzzling lessons from E7 and RET/PTC3 transgenic mice

**DOI:** 10.1038/sj.bjc.6604991

**Published:** 2009-03-17

**Authors:** L Jin, A Burniat, J-E Dumont, F Miot, B Corvilain, B Franc

**Correction to**: British Journal of Cancer (2008) 99, 1874–1883; doi:10.1038/sj.bjc.6604740

During final submission of this paper, the authors very regretfully failed to acknowledge some of the support given to them during the completion of their study. Their full and complete Acknowledgments are given below:

